# Supplementary material for: Health effects of immediate telework introduction during the COVID-19 era in Japan: A cross-sectional study
Source: PLoS One. 2021 Oct 8;16(10):e0256530. doi: 10.1371/journal.pone.0256530 (PMC8500427; doi:10.1371/journal.pone.0256530)
Supplement: S1 File — (DOCX) [file pone.0256530.s001.docx]

**Survey of changes in lifestyle, working ways and health status before and after the emergency statement in Japan due to COVID-19**

Dear participants,

To avoid the spread of COVID-19, the Japanese government declared a state of emergency from April 7 to May 25, and most workers switched from office work to telework. We believe there will be an impact on lifestyle, physical health and mental health as a result of such a drastic change before and after the emergency statement. This study is designed and implemented by the University of Occupational and Environmental Health, Japan and BackTech Inc., to investigate the above issues.

Thank you very much for your support and cooperation in this study. Of course, your personal information is protected and will not be provided to others.

⋇The questionnaire needs approximately 5-10 minutes.

1. Processing of personal information

- Consent to processing of personal information and privacy policy

1. This survey is conducted by the University of Occupational and Environmental Health, Japan and BackTech Inc. Please answer the questionnaire after agreeing to the following research contents.

- Understand the study and agree to assist with the survey

1. Gender

- Male
- Female
- Others

1. Age (numbers only, unit is "years")

【 】

1. E-mail address (This survey may be conducted periodically as the working ways change due to the evolving COVID-19. Please provide your email address if you are willing to cooperate in periodic surveys)

【 】

1. Height (numbers only, unit is "cm")

【 】

1. Where do you currently live?

- Tokyo Metropolis
- Others

1. What is the name of the country you currently live in?

【 】

1. What is your current marital status?

- Married
- Single
- Divorce or death

**Cohabitants**

Please select the suitable choices that apply to your situation

1. Are you currently living with cohabitants?

- Yes
- No

<cohabitants→yes>

1. Who do you live with? (Multiple choices possible)

- Children
- Grandchildren
- Spouse
- Parents
- Grandparents
- Friends
- Boyfriend or girlfriend
- Others【 】

1. [Answer only if you live with your children] Which of the following applies to your youngest child?

- Under 1-year-old
- 1-6 years old
- 7-9 years old
- 10-12 years old
- 13-18 years old
- Over 19 years old

1. What is your spouse's (or cohabitating partner's) current work situation?

- Telework
- Commute to work
- No work

1. You have to miss family activities due to the amount of time you must spend on work responsibilities.

- Strongly agree
- Agree
- Neither agree nor disagree
- Disagree
- Strongly disagree

1. You are often so emotionally drained when you get home from work that it prevents you from contributing to your family.

- Strongly agree
- Agree
- Neither agree nor disagree
- Disagree
- Strongly disagree

1. The behaviors you perform that make you effective at work do not help you to be a better parent and spouse.

- Strongly agree
- Agree
- Neither agree nor disagree
- Disagree
- Strongly disagree

1. You have to miss work activities due to the amount of time you must spend on family responsibilities.

- Strongly agree
- Agree
- Neither agree nor disagree
- Disagree
- Strongly disagree

1. Because you are often stressed from family responsibilities, you have a hard time concentrating on your work.

- Strongly agree
- Agree
- Neither agree nor disagree
- Disagree
- Strongly disagree

1. Behavior that is effective and necessary for you at home would be counterproductive at work.

- Strongly agree
- Agree
- Neither agree nor disagree
- Disagree
- Strongly disagree

**Health**

In the past 30 days, have you ever been to any of the following states? Please select the suitable choices that apply to your situation.

1. Have you felt nervous?

- Not at all.
- Just a little.
- Sometimes.
- Mostly
- Always

1. Did you feel hopeless?

- Not at all.
- Just a little.
- Sometimes.
- Mostly
- Always

1. Did you feel fidgety and restless?

- Not at all.
- Just a little.
- Sometimes.
- Mostly
- Always

1. Did you feel depressed and as if nothing was going to make you feel better?

- Not at all.
- Just a little.
- Sometimes.
- Mostly
- Always

1. Did you find it difficult to do anything?

- Not at all.
- Just a little.
- Sometimes.
- Mostly
- Always

1. Did you feel that you were worthless?

- Not at all.
- Just a little.
- Sometimes.
- Mostly
- Always

1. Please select the item that you felt most stressed about. (If you are not stressed, please select "No pressure.")

- No pressure
- Long working hours
- Worry about employment
- Economic pressure
- Drastic changes in telework
- Work-life balance
- Communication with colleagues
- Communication with cohabitants
- Child support
- Housework
- Personal time reduction
- Personal time increase
- Can't go out for entertainment
- Concerns about health due to COVID-19
- Concerns about health exclude COVID-19
- Do not know

1. Please check the following symptoms if they experienced before and after the emergency statement. ( Multiple choices possible)

| Before the emergency statement   - Asymptomatic - Stiff neck - Eyestrain - Back pain - Fatigue - Feel heavy body - Headache - Diarrhea - Constipation - Dizziness - Tinnitus - Frequent urination - Cough and sputum - Joint pain - Hearing loss - Numb fingers and forearms | After the emergency statement   - Asymptomatic - Stiff neck - Eyestrain - Back pain - Fatigue - Feel heavy body - Headache - Diarrhea - Constipation - Dizziness - Tinnitus - Frequent urination - Cough and sputum - Joint pain - Hearing loss - Numb fingers and forearms |
| --- | --- |

1. What was your weight before the emergency statement? (numbers only, unit is "kg")

【 】

1. Is there any change in weight compared to before the emergency statement?

- Decrease
- No change
- Increase

**Drinking**

Please select the suitable choices that apply to your situation.

1. Did you have a drinking habit before the emergency statement?

- Yes
- No

<drinking habits→yes>

1. How often did you drink alcohol (sake, beer, shochu, etc.) before the emergency statement？

- Every day
- Sometimes
- Hardly drink (cannot drink)

1. Is there any change in frequency of drinking alcohol compared to before the emergency statement？

- Quit drinking
- Decrease
- No change
- Increase

1. How much alcohol did you drink per day before the emergency statement？

- Less than 180ml
- 181~360ml
- 361~480ml
- More than 481ml

**Smoke**

Please select the suitable choices that apply to your situation.

1. Did you have a smoking habit before the emergency statement?

- Yes
- No

<smoking habit→yes>

1. How many cigarettes did you smoke per day before the emergency statement? (Only numbers, unit is "cigarettes/day")

【 】

1. Is there any change in the number of cigarettes you smoke per day compared to before the emergency declaration?

- Quit smoking
- Decrease
- No change
- Increase

**Sleep**

Please select the suitable choices that apply to your situation.

1. How many sleep hours did you get per day before the emergency declaration? (numbers only, unit is "hours")

【 】

1. Is there any change in sleep hours compared to before the emergency declaration?

- Decrease
- No change
- Increase

**Exercise**

Please select the suitable choices that apply to your situation.

1. Did you spend at least one hour per day walking or performing equivalent physical activities before the emergency statement?

- Yes
- No

1. Is there any change in exercise habits compared to before the emergency statement?

- Decrease
- No change
- Increase

<exercise habit→yes>

1. What kind of exercise do you do? (Multiple choices)

- Walking
- Running
- Radio calisthenics
- Stretching
- Muscle training
- Aerobics Dance
- Yoga
- Pilates
- Others【 】

1. How do you do exercise? (Multiple choices)

- Without referring to anything in particular
- YouTube videos
- Internet SNS
- Fitness apps
- Sports games
- Personal trainer's guidance
- Services introduced by the company
- Learned from family or friends
- TV programs
- Magazines
- Others【 】

**Work-related**

Please select the suitable choices that apply to your situation.

1. Which of the following is your occupational category? (Please select the closest one)

- Office Work
- Development & Technology
- Research
- Enterprise Planning
- Customer Support
- Sales & Marketing
- Human Resources and General Affairs
- Customer Service
- Management
- Medical Care Work
- Others【 】

1. What is your current employment status?

- Regular staff
- Contract employee
- Part-time worker
- Temporary employee
- Others【 】

1. Are you currently in a management position?

- Yes
- No

1. (management position→yes) Do you currently have any difficulties in managing your subordinates? If so, please tell us in detail. (Optional Question)

【 】

1. Please select your primary transportation for commuting.

- Public transportation (train, bus, taxi)
- Cars and motorcycles
- Bicycle
- Walking
- Others

1. Which of the following options is closest to your commute time?

- Less than 15 minutes
- 15~30 minutes
- 30~60 minutes
- 60 ~ 90 minutes
- 90~120 minutes
- more than 120 minutes

1. What is the status of your telework implementation?

- Never teleworked
- Started telework due to the emergency statement
- Continuous telework regardless of the emergency statement

<telework→yes>

1. How many days per week do you telework?

| Before the emergency declaration   - 0 - 1-2 - 3-4 - 5 or more | After the emergency declaration   - 0 - 1-2 - 3-4 - 5 or more |
| --- | --- |

1. Are you satisfied with your telework?

- Very satisfied
- Satisfied
- Neither
- Dissatisfied
- Very dissatisfied

1. Is there any change in your working hours during telework compared to before the emergency statement?

- Decrease
- No change
- Increase

1. Is there any change in your meeting hours during telework compared to before the emergency statement?

- Decrease
- No change
- Increase

1. What is the main environment for telework?

- Dedicated workspace
- Dining table
- A simple place (such as a sitting table)
- Others

1. （Answer only if you are sitting in a chair）What type of chair are you using?

- Office chair (with wheels)
- Dining chair (without wheels)
- Stool (without backrest)
- Sofa
- Folding chair
- Legless chair
- Others

1. [Answer only if you have a cohabiter] Do you need to share space with a cohabitant when you are teleworking?

- Yes
- No

1. Please select the items that you felt inconvenient with your telework. (Multiple choices possible)

- No inconvenience
- Not ready of desk environment No information equipment
- No workroom
- Slow Internet speed
- Noise
- Small workspace
- Dim workspace
- No Communication tools Others
