# Supplementary material for: Health effects of immediate telework introduction during the COVID-19 era in Japan: A cross-sectional study
Source: PLoS One. 2021 Oct 8;16(10):e0256530. doi: 10.1371/journal.pone.0256530 (PMC8500427; doi:10.1371/journal.pone.0256530)
Supplement: Attachment — Submitted filename: PONE-D-21-17445-review1-20210627.docx [file pone.0256530.s002.docx]

This study is to identify determine the health effects of teleworking during an emergency statement with COVID-19 in Japan. It is an interesting and important topic in the field of organization behavior. There are some comments as for improving the study.

(1) Introduction: This paper does not demonstrate the importance or value of this work or research. Please demonstrate the theoretical and practical meaning of this work.

(2) Literature review: Insufficient literature review about telework. This paper needs to clear the base of theory, and the between telework, and health factors.

(3) Please add the cited information about measure, such as telework, lifestyle change, and Physical symptoms.

(4) The implications need to improve further.
